# Pelvic Ultrasound Features of Abnormal Uterine Bleeding

**DOI:** 10.2174/0115734056275637260525055216

**Published:** 2026-06-03

**Authors:** Rawan Abdeen, Abdulrahman S. Almalki, Hassan S. Rahmatullah, Salman M. Almadni, Samaher F. Almehmadi, Naglaa Elsayed, Afnan M. F. Darwesh, Fahad Almutairi

**Affiliations:** 1 Department of Radiologic Sciences, Faculty of Applied Medical Sciences, King Abdulaziz University, Jeddah, Saudi Arabia; 2 Department of Radiology, King Abdulaziz University Hospital, Jeddah, Saudi Arabia; 3 Department of Radiology, Faculty of Medicine, Cairo University, Cairo, Egypt

**Keywords:** Abnormal uterine bleeding, Pelvic ultrasound, Ovarian cyst, Fibroids, Gynecology, Menopausal group

## Abstract

**Introduction:**

Abnormal uterine bleeding (AUB) can adversely affect a woman’s life. This condition is experienced by almost 30% of women of reproductive age and nearly 50% of perimenopausal women. This study was conducted to assess the diagnostic value of pelvic ultrasonography in detecting structural causes of abnormal uterine bleeding, and to compare age-related differences between the ultrasonographic findings in early reproductive women, late reproductive women, and menopausal women.

**Materials and Methods:**

In a retrospective study conducted at King Abdulaziz University Hospital, pelvic ultrasound studies were recorded from January 2018 to January 2020. A total of 711 female patients presenting with AUB were examined *via* pelvic ultrasound inspections, with 169 patients meeting the inclusion criteria for this study. The age groups and sonographic findings of these patients were recorded and classified into three groups.

**Results:**

This study demonstrated that ovarian cysts were most common in both the early and late reproductive groups (27.11% and 22.56%, respectively), followed by enlargement of the ovaries in the early reproductive group (12.96%) and enlargement of the uterus in the late reproductive group (22.56%). In the menopausal group, fibroids were the most common finding (24.67%). Despite the high prevalence of ovarian cysts across all groups, the majority were likely benign. However, the presence of uterine enlargement and fibroids warrants further investigation due to the potential clinical implications of these conditions.

**Discussion:**

This study illustrates the applicability and efficacy of pelvic ultrasound procedures in detecting all types of pathologies in the various stages of reproduction. Ovarian cysts and enlargement of the uterus among younger patients and the presence of fibroids in the menopausal cohort were among the high prevalence range of findings well visualized through the use of ultrasound.

**Conclusion:**

This study establishes age-related patterns in ultrasound findings among female patients with AUB. Both the early and late reproductive groups had ovarian cysts, while fibroids were reported to be common in the menopausal group. Knowledge of the age-related distribution of these findings across all stages of the reproductive system may enable sonographers to make informed clinical decisions and enhance patient care.

## 1 INTRODUCTION

Abnormal uterine bleeding (AUB) refers to any alteration in the volume, pattern, or duration of menstrual blood flow [1]. AUB is one of the most prevalent complaints in gynecologic practice, particularly among reproductive-age and perimenopausal women. It is estimated that the prevalence of AUB among women of reproductive age is 10-30%, and 50% among perimenopausal women [2, 3]. Abnormal bleeding is regarded as an indication of uterine disease, which can be acute, as defined by an episode of heavy bleeding requiring immediate intervention, or chronic, as defined by bleeding lasting for at least six months [2]. The bleeding pattern is an important foundation for determining the etiology of AUB and is essential for detecting the condition [4].

AUB can have an incredibly harmful effect on the emotional, physical, and social health of a woman, including her general quality of life. In addition to the direct impact on the women themselves, there is also a loss of productivity in the workplace and a financial cost [5]. Moreover, women with AUB have been reported to consume more health resources than their counterparts without the condition [6]. Thus, it is essential to understand the potential causes of AUB and treat this condition in the correct way. The International Federation of Gynecology and Obstetrics came up with a system to classify structural and nonstructural AUB in order to enable proper diagnosis of the disease and determine the relevant treatment. The categories are classified by the abbreviation PALM-COEIN: polyps (benign tumors that may develop in the lining of the uterus (endometrium)), adenomyosis (a condition in which endometrial tissue proliferates into the uterine muscle wall), leiomyoma (also known as fibroids, which are benign tumors that develop in the uterine muscle wall), malignancy (a rare cause of AUB, although it is important to rule out cancer in any woman experiencing abnormal bleeding), hyperplasia–coagulopathy (a disorder of blood clotting that can cause excessive bleeding), ovulatory disorders (conditions that can lead to irregular or prolonged menstrual bleeding), endometrial conditions (disorders that affect the lining of the uterus and can cause excessive bleeding, spotting between periods, or prolonged periods), iatrogenic causes (issues that stem from medical treatment (some medications, such as anticoagulants and hormonal therapies, can cause irregular bleeding)), and not classified [1].

Ultrasound tests are important for assessing women with AUB. The first modalities of images applied in the clinic or emergency department include transabdominal and transvaginal sonography because they are cheap, non-ionizing, and quite common [7]. Nonetheless, guidance from the Royal College of Obstetricians and Gynecologists (RCOG) recommends transvaginal sonography for women with AUB [8], whereas ultrasound tests are suggested for helping to identify the size, position, texture, and form of the lesion, along with its association with other structures of the pelvis [1]. Combining simple transvaginal scans with the use of three-dimensional ultrasound, Doppler practices, and saline infusion sonohysterography may augment the application of simple scans when indicated, and will generally eliminate the need for invasive diagnostic methods, including hysteroscopy or magnetic resonance imaging [9].

The prevalence and causes of AUB may vary based on age. These variations are examined in this study, which aims to provide useful information on how diagnostic procedures and treatments can be adjusted to particular patient groups. This research proposal focuses on finding the typical structural pathophysiological factors of AUB across various age categories through transvaginal ultrasound investigation with the aim of determining the usefulness of ultrasound as a method of diagnosing various structural defects and characterizing normal situations (as well as the potential underlying pathophysiology) of vaginal bleeding. The current study may establish unique ultrasonic patterns of each cause in order to make a quicker and more accurate diagnosis.

## 2 MATERIALS AND METHODS

This study followed the Declaration of Helsinki and was approved by the Unit of Biomedical Ethics Research Committee at King Abdulaziz University (Reference No 604-21). It was conducted in the Department of Radiology at King Abdulaziz University Hospital in Jeddah, Saudi Arabia. A total of 169 non-pregnant female patients with AUB (ranging in age from 18 to 70 years) were referred by their physicians for pelvic ultrasound examinations. The reasons why 542 patients were not included in the study are numerous, including pregnancy, post-pelvic operation, post-radiotherapy, chemotherapy, being unable to withstand the transvaginal examination, and incomplete information. Despite this, the sample size of 169 participants is adequate enough to reach the objectives of the study while maintaining the appropriate statistical power. The pre-calculated sample size was based on a 20% difference, corresponding to a moderate difference in prevalence between groups, a two-tailed level of the test = 0.05, and a statistical power of 80%. The necessary sample size used to compare the prevalence in the age groups was 150 participants.

Written informed consent was waived because this was a retrospective study. As anonymized imaging and clinical records were retrospectively reviewed, there was no direct contact with the patients involved.

The participants were classified into three groups based on their age: early reproductive (19-30 years old), late reproductive (31-49 years old), and menopausal (50-70 years old). Transvaginal ultrasound examinations were performed using the Philips IU22 machine and a vaginal probe (5-11 MHz). Doppler techniques were also applied when needed.

Patients were placed in the dorsal lithotomy position with an empty bladder. Each scan began by obtaining a longitudinal view of the uterus (Fig. **1A**). From there, the transducer was turned 90 degrees to obtain a transverse scan of the uterus (Fig. **1B**) and demonstrated a good view of the fundus with the endometrium and myometrium. Images of both ovaries were also obtained (Fig. **2**). Doppler techniques were applied, as needed, to obtain the vascularity of uterine or ovarian masses (Fig. **3**) as well as endometrial polyps. Ultrasound findings were then collected and classified according to the participants’ age group.

## 3 STATISTICAL ANALYSIS

Findings and diagnoses were recorded in an MS Excel spreadsheet before being coded and analyzed using Statistical Package for the Social Sciences (SPSS) version 20.0. From there, descriptive statistical analysis was carried out. The data of the findings are presented as both numbers and percentages (%).

Age groups and ultrasonographic findings were compared for their dependence using the Chi-square test. When expected cell frequencies were <5 or zero frequency was present, Fisher’s exact test was used, and a *p*-value of less than 0.05 was considered statistically significant.

## 4 RESULTS

Table **1** demonstrates the distribution of patients with AUB according to age group. Most of the patients were in the late reproductive group at 31-49 years old (51.48%).

Transvaginal ultrasound findings were classified into either normal or abnormal findings, though in some cases, there were additional, associated findings among the three age groups (Table **2**). Normal ultrasound findings were observed in three, eight, and six patients in the early reproductive, late reproductive, and menopausal groups, respectively (5.90%, 6.02%, and 7.80%).

Regarding abnormal findings, Table **3** showed different ultrasound findings in the patients according to their age groups.

In the early reproductive group, ovarian cysts were the most common finding (29.41%) (Fig. **4**), followed by polycystic ovary syndrome (13.72%) (Fig. **5A**, **B**). Fluid in the pouch of Douglas was present in 11.76% of participants, while 9.80% of patients had fibroids (60% of which were submucosal, 20% intramural, and 20% subserosal). Other ultrasonographic findings are presented in Table **3**.

The most common ultrasound findings in the late reproductive group were ovarian cysts and enlargement of the uterus (22.56% for each), followed by fibroids (18.80%), where 64% were subserosal and 36% were intramural. A thickened endometrium was also found in 8.2% of cases. Other ultrasonographic findings are presented in Table **3**.

For the menopausal group, fibroids were the most common finding at 31.17% (where 42.5% were subserosal (Fig. **6A**, **B**), 37.5% submucosal, and 20% intramural), followed by enlargement of the uterus (22.08%), then Nabothian cysts (10.39%). Other ultrasonographic findings are presented in Table **3**. Endometrial polyps (Fig. **7A**) with color Doppler (Fig. **7B**) were found in only two of the participants.

Regarding ovarian cysts (χ^2^ = 13.25, p = 0.001), uterine fibroids (χ^2^ = 9.51, p = 0.009), enlarged uterus (χ^2^ = 6.44, p = 0.040), and polycystic ovary morphology (χ^2^=11.09; p=0.004), there were significant differences between the age groups. Other ultrasound parameters did not demonstrate age-related differences (*p* > 0.05).

Other associated, irrelevant findings are shown in Table **4**. These include cervical blood clots, which were detected in patients with bleeding during examination, as well as uterine abnormalities, including retroflexed and bicornuate uterus syndrome.

Fisher's exact test analysis of the associated ultrasonographic findings did not indicate a statistically significant difference among the age categories (*p* > 0.05 for all comparisons).

## 5 DISCUSSIONS

AUB can be a significant health issue for female patients. The clinical diagnosis of AUB may be difficult; as such, imaging has been identified as useful in reducing the differential diagnosis while ruling out organic pathology [7]. Pelvic ultrasound is the image modality of choice, as it is effective in diagnosing the causes of AUB and also helps physicians and surgeons alike deliver proper treatment and surgical intervention when necessary. According to the results of previous research, the pelvic ultrasound examinations have been reported to show good accuracy with regard to ascertaining atrophy and malignancy and moderate accuracy with regard to ascertaining fibroids and polyps [10]. Ultrasound Doppler techniques play a significant role in ultrasound scans. Some of the features of the ultrasound Doppler methods that are capable of differentiating between benign and malignant fibroids are blood flow pattern, resistance to blood flow, and vascularity [11]. The provided study indicates the flexibility and usefulness of the pelvic ultrasound procedure to detect numerous pathologies at different stages of reproduction. Ovarian cysts and enlargement of the uterus among the younger age group and fibroids in the menopausal cohort, were only among the high prevalence range of findings well visualized through the use of ultrasound. This further adds weight to the notion that ultrasound is a useful and effective instrument when it comes to the complete evaluation of the pelvis and early identification of possible anomalies.

Most of the patients in this study were of late reproductive age (39-49 years old) with an average age of 40.52 years, accounting for 51.48% of the total cases. The obtained results are in line with the results of the study carried out by Daga and Phatak, who detected that the majority of patients with AUB were in the 40-45 age range (representing 64.63%) [1]. The same results were obtained in another study, where the 40-45 age group showed the highest rates of AUB [3]. This study indicates that the most prevalent diagnosis that patients with AUB have regarding the early and late reproductive age groups is ovarian cysts, which often present with no symptoms.

There are many types of ovarian cysts, with the most common being functional cysts. These cysts are brought about by natural changes of the ovaries in the menstrual cycle. They tend to be harmless and clear up on their own in a matter of weeks with no complications [12]. Follicular cysts (when the follicle has failed to release an egg during ovulation) and corpus luteum cysts (when the follicle that released an egg upon ovulation fills with fluid) are two typical examples of functional cysts [13]. Polycystic ovary syndrome is another type of ovarian cyst that causes irregular periods, weight gain, and hirsutism [13]. The risk of torsion that accompanies each type of ovarian cyst is different. Benign cysts include functional cysts (e.g. follicular cysts and corpus luteum cysts). They do not twist as easily compared to other forms of cysts. Risk of torsion is also determined by the size of the cyst. For example, bigger cysts have more chances of twisting as compared to small cysts. Functional cysts occur at a low risk of torsion, which is 1-2%. A cyst with a diameter of above 5 cm is more inclined to twist in comparison with a smaller cyst. Cysts located on the outside of the ovary are also more likely to cause torsion [14]. The mean size of ovarian cysts was 3.5 x 3.5 cm, as shown in the current study, the presence of the cysts is a known cause of abnormal vaginal bleeding. The findings of the research are in accordance with the findings by Conyne and Smith, who have proven that large cysts may lead to pain in the pelvis and vaginal bleeding [15]. In addition, vaginal bleeding may happen with functioning ovarian cysts. It has been reported that ultrasound tests were found to be of good sensitivity and specificity with respect to the diagnosis of ovarian cysts [16]. This study also draws attention to the fact that ovarian results are dynamic in various stages of reproduction. Although ovarian cysts were the most commonly observed condition in all the groups, as age increased, the prevalence became less common, and fibroids took over in the menopausal group. Increased ovaries and uterus size were also found, especially among the younger reproductive age group. These results highlight the need to pay attention to the reproductive stage during the interpretation of pelvic ultrasound results and design screenings or intervention plans in accordance with the results.

In the present study, uterine fibroids were the most common finding in the menopausal group and the third most common finding in the late reproductive group. A previous study reported that fibroids were the predominant organic major cause for AUB within the PALM-COEIN classification system [1]. Uimari *et al*. reported that uterine fibroids impact millions of women globally, with a 75% prevalence among women of reproductive age. Uterine fibroids cause excessive menstrual bleeding (or menorrhagia) in about 30% of patients, while more than 50% of patients experience symptoms, including pelvic pain, infertility, and heavy uterine bleeding, which can lead to severe anemia and negatively affect one’s quality of life [17]. Regarding the literature on fibroids, there is no universal agreement on the mechanisms of uterine bleeding secondary to fibroids. It is posited that uterine fibroids induce irregular menstrual bleeding by modifying both local and distant endometrial gene expressions, hence affecting endometrial function [18].

Nabothian cysts were a common finding among the three patient groups in this study. These mucus-filled cysts can be found on the surface of the uterine cervix. Nabothian cysts are usually benign lesions that appear as firm bumps [19]. Kim *et al*. reported that the occurrence of Nabothian cysts is usually associated with the inflammatory and reparative process of chronic cervicitis or due to minor trauma (often after childbirth) [20]. Most Nabothian cysts are small in size. However, a large cyst can sometimes block the cervical opening and cause irregular vaginal bleeding [19].

The best way to assess the endometrium is through ultrasound, which enables an accurate visualization and measurement of the endometrium [3]. Endometrial thickness has different interpretations based on the menstrual cycle phase and age. Thickness was measured on the outer margin of the anterior endometrial-myometrial junction to the outer margin of the posterior junction in the sagittal section. In the case of postmenopausal patients, the endometrial thickness was deemed to be thick when it was greater than 4 mm. Among the early and late reproductive patients, the phase of the menstrual cycle is also significant in establishing the thickness. The preovulatory endometrium is typically 4 to 8 mm, but the postovulatory endometrium is typically 7 to 14 mm [21]. Anything larger than these limits was categorized as thickened. A thickened endometrium was found more in the late reproductive and menopausal cohorts. This was also comparable to another study carried out by Talukdar and Mahela, who discovered that 11.65% of premenopausal women with AUB were found to have a thickened endometrium on ultrasound findings [22]. A thick endometrial tissue can be a good sign of endometrial pathology, especially in older women [22]. The possible cause is that the patient might be having endometrial hyperplasia, which is a common ailment brought about by exogenous or endogenous exposure to estrogen and a relative deficiency in progesterone. Endometrial hyperplasia may lead to irregular thickening of the uterine lining, particularly for women in the menopausal group [8].

Endometrial polyps are a frequent gynecological pathology, which leads to local abnormal overgrowth of the endometrial lining of the stroma and endometrial glands [9]. They have been established previously to vary between 7.8% and 34.9%. This research discovered endometrial polyps only among patients in the menopausal age group. It has been indicated that age is a risk factor for the occurrence of endometrial polyps, which was also the case with this study. Indeed, 20% of AUB in postmenopausal women is attributable to the condition, whereas this is true for only 1% of AUB cases in women aged below 30 years [9]. A previous study assessing endometrial polyps through transvaginal ultrasound examination noted that endometrial polyps were usually linked to AUB [23]. The ultrasonographic results in the current study are presumed pathologies and not a certain diagnosis. More implicated investigations that include the correlation of histopathology are necessary to enhance diagnostic quality and elucidate the actual clinical significance of these ultrasound observations.

There are other endometrial causes of AUB, such as endometrial carcinoma, arterial venous malformations, and gestational trophoblastic tumors, which are important, but nevertheless were not discovered in the present study. In such situations, proper diagnosis is possible with ultrasound, whereas other diagnostic parameters are absent in certain outpatient clinics, such as serum beta HCG (Human Chorionic Gonadotropin) as an indicator of gestational trophoblastic tumors [24]. The abnormalities detected in ultrasound readings are not related to the clinical history of the participants who displayed abnormal bleeding. As an example, larger uteruses were prevalent among the late reproductive and menopause populations. Uterine size interpretation was made on the basis of the standard normative measurement criteria, which were published by Hassan et.al; according to which the mean uterine length, width, and thickness were 7.33 ± 0.76 cm, 3.93 ± 1.00 cm, and 3.44 ± 0.55 cm [25]. In this study, enlargement of the uterus was determined as a size that was above this normative reference range. The increased rate of uterus enlargement among this group of people can signify the physiological alterations that commonly lead to the development of this condition, instead of pathological development. It is important to differentiate the normative finding and the pathological enlargement to avoid unnecessary interventions. This observation contradicts the statistics of Hamd, who concluded that the average uterine size declines with the age of a woman [26]. The prevalence patterns of ages would potentially help clinicians and sonographers differentiate diagnostics. The awareness of these trends can be used to make improved clinical decisions in patients with AUB by utilizing more focused ultrasound scans. Inclusion of age-specific expectations in daily scanning and interpretation might increase diagnostic efficiency.

Fluid in the pouch of Douglas is likely due to the scan being conducted during ovulation. Meanwhile, the presence of a bicornuate uterus or retroverted uterus is an incidental finding that is unrelated to abnormal vaginal bleeding. Normal ultrasound findings were detected in each age group (more in older patients). We suggest that the cause of bleeding in the presence of normal US findings may be referred to as a functional/hormonal abnormality rather than structural changes.

While this study provides valuable insights into pelvic ultrasound findings across different reproductive stages, further research is needed to elucidate the specific factors contributing to observed changes. Investigating the hormonal dynamics and underlying processes associated with ovarian cysts, uterine enlargement, and fibroids could offer a deeper understanding of their development and potential health risks. Additionally, studies exploring the effectiveness of targeted interventions based on reproductive stage could improve clinical management and patient outcomes.

## 6 STUDY LIMITATION

This study has several limitations. The main limitation is a lack of histo-pathologic correlation with ultrasound findings as well as the retrospective design, which limits the causal inference and diagnostic validation. The ultrasound scans performed by practitioners with different levels of experience produce the potential for inter-observer variability. Moreover, selection bias might exist, as this is a single-center study, which limits its generalizability to others. Furthermore, the current data is linked to structural causes of AUB, while non-structural causes are not measured. Therefore, a comprehensive analysis of all components of the PALM-COEIN classification might not be achieved.

## CONCLUSION

This study offers compelling evidence for the crucial role of pelvic ultrasound in safeguarding pelvic health across the female lifespan. The study defines age-related outlines in ultrasound images among female patients diagnosed with AUB. Ovarian cysts were most common in both the early and late reproductive groups, while fibroids were noted to be more prevalent in the menopausal group. Understanding the age-specific distribution of these findings across reproductive stages can help sonographers with clinical decision-making, improve patient care, and enhance quality of life. Future studies should use a prospective design that incorporates both structural and nonstructural causes of abnormal uterine bleeding as classified by the PALM-COEIN system, along with histopathological correlation for more comprehensive evaluation of diagnostic accuracy.

## Figures and Tables

**Fig. (1):**
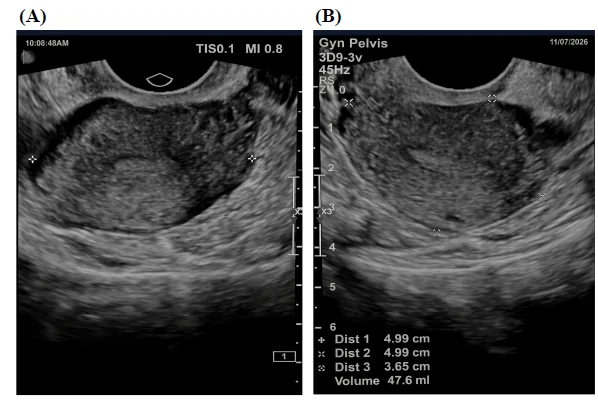
Transvaginal Ultrasound of uterus. **A**: Longitudinal view, **B**: Transverse view.

**Fig. (2):**
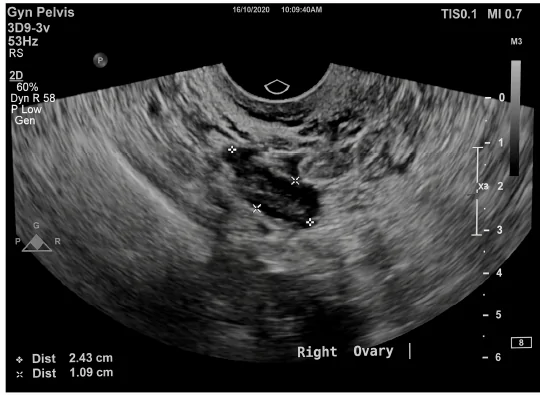
Transvaginal Ultrasound of the left ovary.

**Fig. (3):**
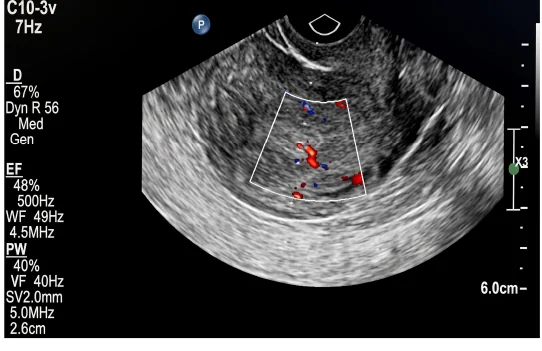
Transvaginal US with Color Doppler application showing normal uterine and endometrial vascularity.

**Fig. (4):**
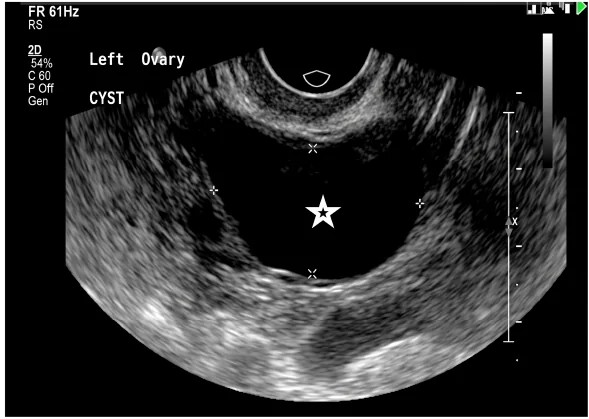
Ultrasound image of left ovary showing large, clear, thin-walled cyst (star).

**Fig. (5A, B):**
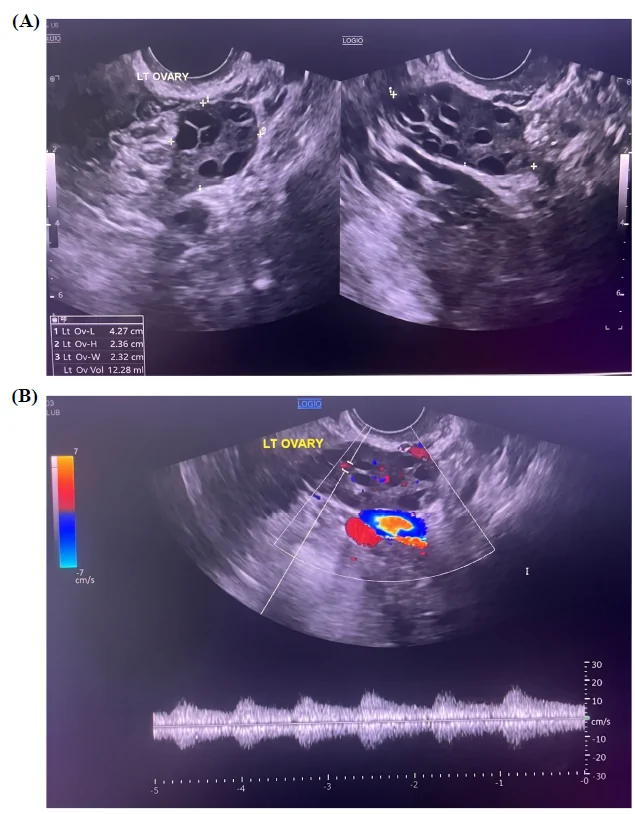
Ultrasound image of left ovary showing enlarged ovary with multipole peripheral cyst demonstrating polycystic ovarian syndrome.

**Fig. (6A, B):**
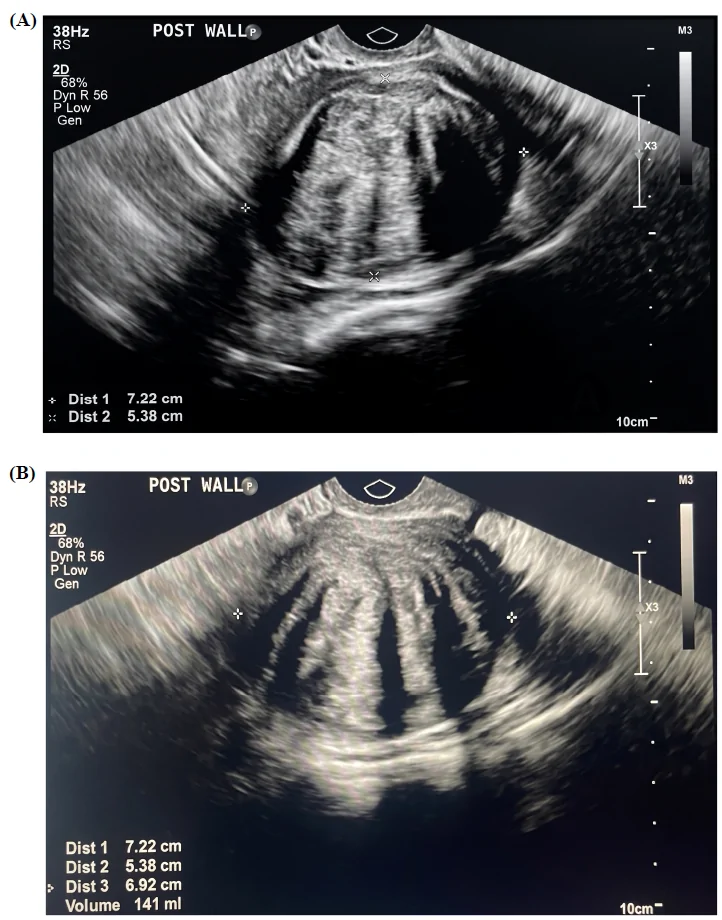
Transvaginal ultrasound image of large subserosal fibroid measuring 7.2X 5.3 X 5 cm.

**Fig. (7A, B):**
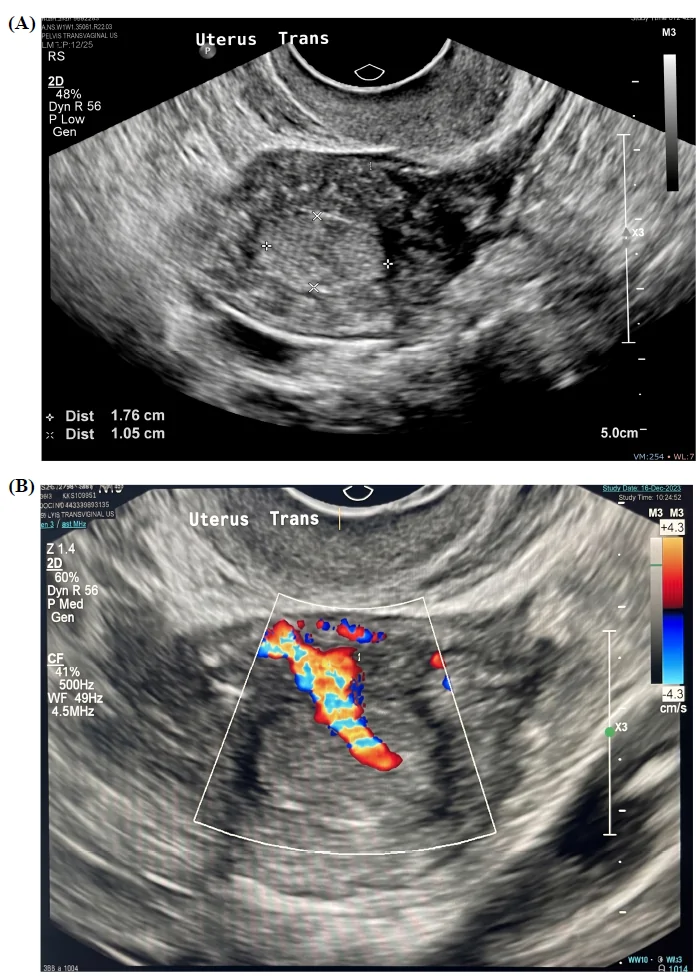
Ultrasound image of endometrial polyps with color doppler.

**Table 1:** Distribution of patients according to their age group.

**Group** **(Age)**	**Number of Patients** **(Number, %)**	**Mean Age (Year)**
Early reproductive (19 - 30)	42 (24.85)	25.90
Late reproductive (31 - 49)	87 (51.48)	40.52
Menopausal (50 - 70)	40 (23.67)	56.10

**Table 2:** Summary of transvaginal ultrasound findings in patients with abnormal uterine bleeding.

Ultrasonographic Findings	**Early Reproductive Number of Cases, (%)**	**Late Reproductive Number of Cases, (%)**	**Menopausal Number of Cases, (%)**
Normal examination	3 (5.9)	8 (6.02)	6 (7.80)
Abnormal findings	43 (84.30)	124 (93.23)	68 (88.30)
Associated findings	5 (9.80)	1 (0.75)	3 (3.90)
**Total number of cases**	51	133	77

**Table 3:** Abnormal transvaginal ultrasound findings in different age groups with abnormal uterine bleeding.

Ultrasonographic Findings	**Early Reproductive** **Number of Cases (%)**	**Late Reproductive** **Number of Cases (%)**	**Menopausal** **Number of Cases (%)**	**χ^2^**	***p*-value**
Ovarian cyst	15 (29.41)	30 (22.56)	5 (6.50)	13.25	0.001
Fibroid	5 (9.80)	25 (18.80)	24 (31.17)	9.51	0.009
Enlargement of uterus	3 (5.88)	30 (22.56)	17 (22.08)	6.44	0.040
Polycystic ovary	7 (13.72)	9 (6.77)	0 (0)	11.09	0.004
Fluid in pouch of Douglas	6 (11.76)	8 (6.02)	2 (2.59)	5.09	0.079
Nabothian cyst	4 (7.84)	10 (7.52)	8 (10.39)	0.71	0.702
Ovarian mass	1 (1.96)	0 (0)	0 (0)	4.48^†^	0.106
Heterogeneous texture of the uterus (adenomyosis)	2 (3.92)	1 (0.75)	5 (6.50)	5.97^†^	0.051
Thickened endometrium	0 (0)	11 (8.27)	5 (6.50)	4.00^†^	0.135
Endometrial polyp	0 (0)	0 (0)	2 (2.59)	4.95^†^	0.084
**Total number of cases**	43	124	68		

**Notes:** Numbers (%) are used to display data. The Chi-squared statistic is represented by χ^2^ values. ^†^Fisher's exact test was used for results with zero values or small expected cell counts; corresponding *p*-values are provided. The threshold for statistical significance was set at *p* < 0.05.

**Table 4:** Transvaginal ultrasound associated findings in different age groups with abnormal uterine bleeding.

**Associated Findings**	**Early Reproductive** **Number of Cases (%)**	**Late Reproductive** **Number of Cases (%)**	**Menopausal** **Number of Cases (%)**	**χ^2^**	***p*-value**
Cervical blood clot	3 (5.88)	0 (0)	0 (0)	5.41^†^	0.067
Bicornuate uterus	2 (3.92)	1 (0.75)	0 (0)	1.68^†^	0.432
Retroflexed uterus	0 (0)	0 (0)	3 (3.89)	5.87^†^	0.053
**Total number**	**5**	**1**	**3**		

**Notes:** ^†^Fisher’s exact test was applied due to small expected cell counts. χ^2^ values are shown descriptively. A *p*-value < 0.05 was considered statistically significant.

## Data Availability

The data that support the findings of this study are available from the corresponding author [R.A] upon reasonable request.

## LIST OF ABBREVIATIONS

AUB: Abnormal uterine bleeding
PALM-COEIN: Polyp; Adenomyosis; Leiomyoma; Malignancy And Hyperplasia; Coagulopathy; Ovulatory Dysfunction; Endometrial; Iatrogenic; and not yet classified
RCOG: Royal College of Obstetricians and Gynecologists
SPSS: Statistical Package for the Social Sciences
Fig: Figure
PCOS: Polycystic ovary syndrome
US: Ultrasound

## STANDARDS OF REPORTING

STROBE guidelines were followed.
